# Clinical impact of a structured secondary cardiovascular prevention program following acute coronary syndromes: A prospective multicenter healthcare intervention

**DOI:** 10.1371/journal.pone.0211464

**Published:** 2019-02-21

**Authors:** David Carballo, Nicolas Rodondi, Reto Auer, Sebastian Carballo, David Nanchen, Lorenz Räber, Roland Klingenberg, Pierre-Frédéric Keller, Dik Heg, Peter Jüni, Olivier Muller, Christian M. Matter, Thomas F. Lüscher, Stephan Windecker, Francois Mach, Baris Gencer

**Affiliations:** 1 Cardiology Division, Geneva University Hospitals, Geneva, Switzerland; 2 Department of General Internal Medicine, University Hospital of Bern, Bern, Switzerland; 3 Institute of Primary Health Care (BIHAM), University of Bern, Bern, Switzerland; 4 Department of Ambulatory Care and Community Medicine, Lausanne University, Lausanne, Switzerland; 5 Department of Internal Medicine, Geneva University Hospitals, Geneva, Switzerland; 6 Department of Cardiology, University Hospital of Bern, Bern, Switzerland; 7 Department of Cardiology, University Heart Center, University of Zurich, Zurich, Switzerland; 8 Institute of Social and Preventive Medicine, and Clinical Trials Unit, Department of Clinical Research, University of Bern, Bern, Switzerland; 9 Director, Applied Health Research Centre (AHRC), The HUB, Li Ka Shing Knowledge Institute, St. Michael’s Hospital, Toronto, Canada; 10 Cardiology Division, Lausanne University, Lausanne, Switzerland; Kurume University School of Medicine, JAPAN

## Abstract

**Background:**

Structured secondary cardiovascular prevention programs (SSCP) following acute coronary syndromes (ACS) may reduce major adverse cardiovascular events (MACE) through better adherence to post-ACS recommendations.

**Methods:**

Through a prospective multicenter cohort study, we compared the outcomes of two sequential post-ACS patient cohorts, the initial one receiving standard care (SC) followed by one receiving additional interventions (SSCP) aimed at improving patient education as well as healthcare provider and hospital systems. The primary endpoint was MACE at one year. Secondary endpoints included adherence to recommended therapies, attendance to cardiac rehabilitation (CR) and successful achievement of cardiovascular risk factor (CVRF) targets.

**Results:**

In total, 2498 post-ACS patients from 4 Swiss university hospitals were included: 1210 vs 1288 in the SC and SSCP groups, respectively. The SSCP group demonstrated a significant increase in attendance to CR programs (RR 1.08, 95%CI 1.02–1.14, P = 0.006), despite not achieving the primary MACE endpoint (HR 0.97, 95%CI 0.77–1.22, P = 0.79). After age-stratification, significant reductions in cardiac death, MI and stroke events (HR 0.53, 95%CI 0.30–0.93, P for interaction = 0.016) were observed for SSCP patients ≤ 65 years old. The SSCP group also scored significantly better for the LDL cholesterol target (RR 1.07, 95%CI 1.02–1.13, P = 0.012), systolic blood pressure target (RR 1.06, 95%CI 1.01–1.13, P = 0.029) and physical activity (RR 1.10, 95%CI 1.01–1.20, P = 0.021).

**Conclusions:**

The implementation of an SSCP post ACS was associated with an improvement in the control of CVRF and attendance to CR programs, and was also associated with significant reductions in cardiac death, MI and stroke at one year for patients ≤65years old.

## Introduction

The prognosis of acute coronary syndromes (ACS) has considerably improved in recent years with the implementation of recommended post-ACS therapies.[1] In this regard, a better understanding of therapeutic strategies resulting from evidence-based clinical research has led to improvements in patient long-term medication compliance and clinical outcomes.[2] In 2012, the European Society of Cardiology (ESC) issued guidelines recommending the implementation of national programs at the hospital level to monitor the quality of care of ACS patients, as well as the development of multidimensional programs based on motivational interviewing.[3] In addition, participation in cardiac rehabilitation (CR) programs following hospital discharge is strongly recommended to improve patient lifestyle and long-term prognosis post-ACS.[4, 5] Despite all these measures, recent European observational data for ACS patients still point to poorly controlled targets, resulting in suboptimal reductions in the incidence of major adverse cardiovascular events (MACE) and participation in CR programs.[6, 7]

In Europe, few studies have tested secondary prevention intervention programs aimed at improving post-ACS cardiovascular outcomes. The EUROACTION study group ran a nurse-coordinated, multidisciplinary, family-based, ambulatory prevention program for patients with coronary heart disease (CHD). The program highlighted both the beneficial effects of a healthier lifestyle, and demonstrable improvements in cardiovascular risk factor control.[6] Improving quality of care is especially important towards reducing the burden of healthcare costs associated with ACS patients, and also enables hospitals to meet benchmark criteria.[8, 9] Switzerland recently implemented a hospital payment system based on diagnostic-related groups (DRGs), aiming among other things at being able to objectively compare healthcare provision across hospitals.[10]

A meta-analysis previously conducted by our group showed that in-hospital, in-patient interventions in secondary prevention could lead to a reduction in mortality, although the evidence was not definitive.[11] Based on these findings, we designed a structured secondary cardiovascular prevention program (SSCP), named ELIPS, a multi-dim**E**nsiona**L** prevent**I**on **P**rogram after Acute coronary **S**yndromes, aiming at improving the quality of care of patients post-ACS and thereby reducing related mortality and morbidity.

## Methods

### Study population

ELIPS (NCT01075867) (Protocole 07–131) is part of a collaborative research project (Inflammation and acute coronary syndromes (ACS)–Novel strategies for prevention and clinical management) supported by the Swiss National Science Foundation in 4 Swiss university hospitals (Bern, Geneva, Lausanne and Zurich). Subjects ≥ 18 years old hospitalized with a main diagnosis of ACS were recruited from January 2009 to December 2012. ACS were defined as symptoms compatible with angina pectoris (chest pain, breathlessness) and at least one of the following criteria: (a) ECG ischemic changes, such as persistent or dynamic ST-segment deviation, T-waves inversion, new left bundle branch block; (b) evidence of positive conventional or high-sensitive troponin by local laboratory reference values; (c) known coronary heart disease (CHD) defined by pre-existing myocardial infarction (MI), coronary artery bypass graft (CABG), percutaneous coronary intervention (PCI), or ≥50% documented stenosis of an epicardial coronary artery in a previous angiography.[12] Exclusion criteria comprised index revascularization with CABG, severe physical disability, inability to give consent (dementia) and less than 1 year of life expectancy for non-cardiac reasons. To evaluate the effectiveness of the ELIPS intervention, we chose a prospective sequential before-after intervention design, as is usual for complex interventions.[11] The standard care (SC) group (observation phase) comprised patients enrolled from January 2009 to December 2010, while the ELIPS add-on group (intervention phase) comprised patients enrolled from January 2011 to December 2012. The centralized institutional review board (Comité Départemental d’Ethique de Médecine Interne et Médecine Communautaire, Hôpitaux Universitaires de Genève) approved the protocol (Protocole 07–131) on August 27^th^, 2007, and all participants gave written informed consent.

### Description of the ELIPS intervention

The ELIPS intervention includes actions at the patient, healthcare provider and healthcare system levels aimed at improving patient outcomes through better adherence to post-ACS CR programs.[13, 14]

#### ELIPS at the patient level

At the patient level, ELIPS consisted of a CR educational program delivered to the patient at appropriate timelines and intervals, based on individually defined needs during the post-ACS hospitalization period. Patients were encouraged to achieve a healthy lifestyle with the support of health professionals trained in motivational interviewing.[15] Motivational interviewing is a non-judgmental, patient-centered counselling approach aimed at eliciting and strengthening the motivation to change.[16] Patients were asked to self-evaluate their cardiovascular risk factors with the aid of an interactive wall chart and invited to watch a 27-minute film (provided in the form of a DVD) portraying the real life trajectory of a patient suffering from ACS. Patients also received personalized educational lifestyle brochures and were discharged with a standardized treatment discharge card detailing the reasons for their prescriptions, as well as a summary of their targets in secondary prevention. Patient’s comprehension of point-by-point educational content was not formally evaluated. A dedicated website (https://www.hug-ge.ch/elips) enabled both patients and healthcare providers to remain up to date with the therapeutic education process and training program. Phase 2 cardiac rehabilitation and exercise prescription was carried out in nationally registered cardiac rehabilitation centers following hospitalization discharge.

#### ELIPS at the healthcare provider level

At the healthcare provider level, dedicated nurses at each of the 4 hospitals were trained in motivational interviewing and cardiovascular health education by certified nurse specialists (Motivational Interviewing Network of Trainers). Interviews were centred around the patient in order to resolve any ambivalent motivation while at the same time reinforcing intrinsic motivation for change.[15]

#### ELIPS at the system level

At the system level, project leaders at each participating hospital were responsible for organizing a series of educational sessions to support the implementation of the ELIPS intervention. The aforementioned standardized treatment discharge cards were aimed at relaying follow-up information to family physicians or designated cardiologists.

As is described, the ELIPS multifaceted intervention was a program with incentives for therapeutic adherence and lifestyle modification without pharmacologic or invasive interventions. As such, it may not qualify as a clinical trial. In order to ensure, however, a public record of the study, reduce publication bias and complete fulfilment of ethical obligations towards the participants, the study was registered with the National Institutes of Health U.S. National Library of Medicine ClinicalTrials.gov registry. This registration process was initiated during the first months of the SPUM-ACS (Special Program University Medicine—Acute Coronary Syndrome, NCT01075867), after initial participant enrollment, which began in January 2009; the registration was first posted on February 25^th^, 2010. The authors confirm that all ongoing and related trials for this intervention are registered.

### Study outcomes

The primary endpoint of MACE at one-year was a composite of death from any cause, recurrence of MI, unplanned coronary revascularization, hospitalization for unstable angina, lower limb ischemia and stroke events. All clinical endpoints were adjudicated by a panel of independent experts (three certified cardiologists) blinded to the allocation groups. Secondary endpoints included the documentation of recommended therapies (such as aspirin, statins, beta-blockers or angiotensin converting enzyme (ACE) inhibitors/angiotensin receptor blockers (ARB)) at discharge and at one-year follow-up.[17] Given the controversies over what the optimal treatment duration of P_2_Y12 inhibitors should be, we discarded this point from endpoint analyses. The level of attendance to CR programs was assessed based on data collected at discharge (direct transfer) and at one-year follow-up, where patients were asked if they attended a CR program (in-patient or out-patient). Recommended secondary preventive targets at one year were defined as low-density lipoprotein (LDL) cholesterol < 1.8 mmol/l (70 mg/dl), systolic blood pressure < 140 mm Hg, fasting plasma glucose < 7 mmol/l in non-diabetic patients, glycated haemoglobin < 7% in diabetic patients, and weight reduction of ≥ 5% in overweight (body mass index [BMI] 25.0–29.9 kg/m^2^) or obese (≥ 30.0 kg/m^2^) subjects.[6] For behavioural outcomes, targets were defined as smoking cessation in smokers (based on 7-day point prevalence rates), high medication adherence (defined by a score of zero using the Morisky Medical Adherence Scale (MAS)), health utility index (based on the Euroqol-5 dimensions (EQ-5D)), and level of physical activity (defined by at least 3 days/week vigorous-intensity activity or 5 days/week moderate-intensity activity, according to the international physical activity questionnaire (IPAQ)).

### Statistical analysis

Data were expressed as medians ± interquartile range (IQR) for continuous variables, and as numbers and percentages for categorical variables. Time-to-first event or composite events were analysed censoring patients at 365 days, at death or last valid contact date. The association between the ELIPS intervention and the primary endpoint was expressed with hazard ratios (HR) and 95% confidence intervals (CI) using Cox regression proportional hazards. In the multivariate model, we adjusted for age and sex. The association with one-year preventive targets’ achievement was expressed with rate ratios (RR), using the method of generalized linear regression with a log link function and Poisson pseudo-maximum likelihood estimator. For the sample size calculation, the estimated incidence of MACE at one-year was 15% for the control group.[18] Based on our previous meta-analysis, we hypothesized an absolute risk reduction of 4% for the ELIPS add-on intervention group.[11] Assuming a 2-sided alpha level of 0.05 (5% level of significance) and a power of 0.80, it was estimated that a sample size of 1158 patients per group was required in order to detect the expected effect size. To account for potential dropouts, our overall sample size was increased by 4% to 2400 ACS patients. Statistical analyses were performed using the STATA statistical software (Version 13, STATA Corp, College Station, TX, USA).

## Results

**Fig 1:**
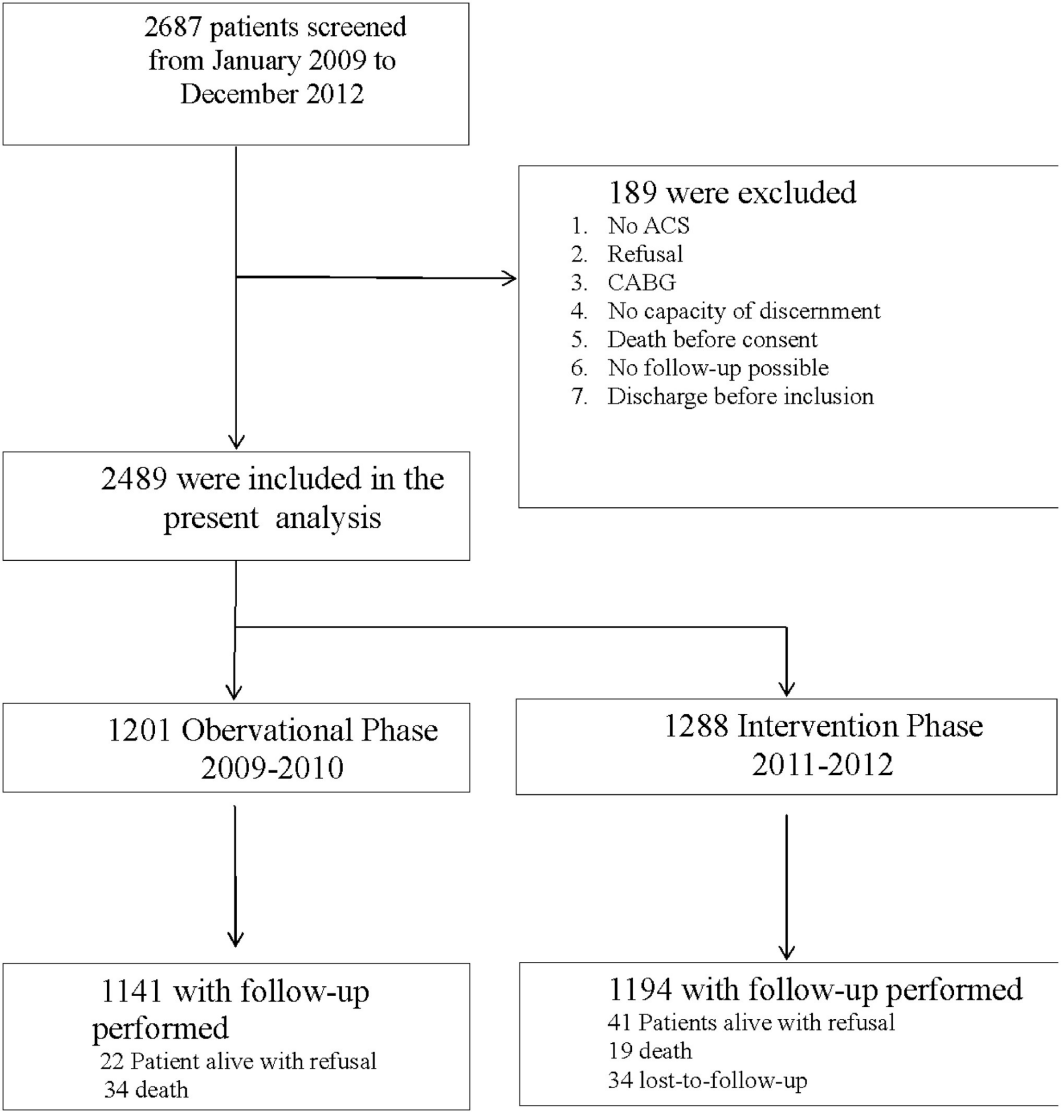
Flowchart of the studied cohort.

A total of 2498 patients were included, 1210 in the SC group (2009–2010) and 1288 in the ELIPS add-on group (2011–2012) (Fig 1). Mean age was 62.3±12.3 (the age distribution is illustrated in S1 Fig), 21.1% were women, 54.7% had STEMI, 40.2% NSTEMI and 5.0% unstable angina (Table 1). The reported use of educational ELIPS tools by healthcare providers was as follows: 68.6% for motivational interviewing, 54.5% for the wall chart, 68.7% for the use of educational brochures, 74.2% for the film provided on DVD, 52.7% for the website and 82.8% for the discharge medication card.

**Table 1:** Baseline characteristics of 2498 participating ACS patients.

Variables	Standard Care 2009–2010 N = 1210	ELIPS add-on 2011–2012 N = 1288	P value
**Socio-demographic**			
Age (years), mean ± SD	63.6 ± 12.6	61.2 ± 11.9	< 0.001
Women, n (%)	255 (21.1)	271 (21.0)	0.98
BMI (kg/m^2^), mean ± SD	27.1 ± 4.2	27.1 ± 4.3	0.74
High education level, n (%)	174 (15.0)	186 (14.8)	0.87
**Medical history**			
Diabetes, n (%)	215 (17.8)	208 (16.2)	0.28
Hypertension, n (%)	699 (57.8)	681 (52.9)	0.013
Hypercholesterolemia, n (%)	713 (59.1)	778 (60.6)	0.44
Current smoker, n (%)	462 (38.3)	551 (42.8)	0.03
Previous MI, n (%)	209 (17.3)	178 (13.8)	0.017
Previous Stroke, n (%)	33 (2.7)	27 (2.1)	0.30
**CV treatment prior ACS**			
Aspirin, n (%)	378 (31.2)	358 (28.0)	0.074
Statins, n (%)	372 (30.7)	354 (27.7)	0.095
Beta-blockers, n (%)	267 (22.1)	272 (21.3)	0.63
ACE inhibitors/ARB, n (%)	429 (35.5)	415 (32.6)	0.12
**Hospital data**			
ACS diagnosis			
STEMI, n (%)	639 (52.9)	725 (56.4)	0.020
NSTEMI, n (%)	510 (39.7)	493 (40.1)	
Unstable angina, n (%)	51 (4.0)	75 (6.2)	
Killip at admission			
Class I, n (%)	1040 (88.8)	1136 (88.6)	0.21
Class II-IV, n (%)	131 (11.2)	146 (11.4)	
Index revascularization			
PCI with stent, n (%)	1028 (84.9)	1134 (88.0)	0.009
PCI without stent, n (%)	60 (5.0)	67 (5.2)	
Conservative, n (%)	123 (10.2)	87 (6.8)	
Length of stay, mean (±SD)	4.9 ± 5.3	4.2 ± 3.9	0.006

Abbreviations: ACE, angiotensin converting enzyme; ACS, acute coronary syndrome; ARB, angiotensin receptor blocker; BMI, body mass index; CV, cardiovascular; NSTEMI, Non ST-elevation myocardial infarction; PCI, percutaneous coronary intervention; SD, standard deviation; STEMI, ST-elevation myocardial infarction. Missing values: 24 for BMI, 74 for education, 1 for diabetes, 1 for hypertension, 2 for smoking, 3 for previous MI 5 for ACS diagnosis, 8 for aspirin, 10 for statin, 13 for beta-blockers, 15 for ACE inhibitor or ARB, 45 for Killip

Except for the use of beta-blockers, no significant differences were found between the SC and ELIPS add-on groups regarding the prescription of recommended medications at discharge, or reported use of the said medications at one year (Table 2). The use of beta-blockers was higher at one year in the ELIPS add-on group (80.2% vs. 76.0%, RR 1.05 95% CI 1.01–1.10, P = 0.019). The rate of participation in a CR program was also significantly higher in the ELIPS add-on arm (72.9% vs. 65.6%, RR 1.08, 95% 1.02–1.14, P = 0.006).

**Table 2:** Recommended therapies at discharge and at one year in standard care vs. ELIPS add-on groups.

Process Outcomes	Standard Care 2009–2010 N = 1210	ELIPS add-on 2011–2012 N = 1288	Age-sex adjusted Rate Ratios (95% CI)	*P-*value
**Aspirin**				
Prescription at discharge, n (%)	1201 (99.4)	1268 (99.1)	1.00 (0.99–1.00)	0.32
Reported use at one year, n (%)	1098 (96.9)	1163 (97.2)	1.00 (0.99–1.01)	0.95
**Statins**				
Prescription at discharge, n (%)	1182 (97.8)	1259 (98.5)	1.01 (0.99–1.02)	0.35
Reported use at one year, n (%)	1060 (93.6)	1101(92.1)	0.98 (0.96–1.00)	0.12
**Beta-blockers**				
Prescription at discharge, n (%)	1015 (83.9)	1083 (84.1)	1.00 (0.97–1.04)	0.82
Reported use at one year, n (%)	861 (76.0)	959 (80.2)	1.05 (1.01–1.10)	0.019
**ACE inhibitors or ARBs**				
Prescription at discharge, n (%)	1104 (91.3)	1159 (90.7)	0.99 (0.97–1.02)	0.57
Reported use at one year, n (%)	917 (80.9)	980 (82.0)	1.02 (0.98–1.06)	0.37
**P**_**2**_**Y12 inhibitors**				
Prescription at discharge, n (%)	1140 (99.9)	1224 (99.0)	0.99 (0.98–1.00)	0.001
Reported use at one year, n (%) *	889 (97.5)	905 (81.0)	0.83 (0.80–0.86)	< 0.001
**Attendance to CR after discharge, n (%)**	730 (65.6)	860 (72.9)	1.08 (1.02–1.14)	0.006

Abbreviations: ACE, angiotensin converting enzyme; ARB, angiotensin receptor blocker; CI, confidence intervals; CR, cardiac rehabilitation. Missing values at discharge: 11 for aspirin, 12 for statins, 13 for beta-blockers, 12 for ACE inhibitors/ARBs and 120 for P_2_Y12 inhibitors. Missing values at one year (appropriate or not): 169 for aspirin, 170 for statins, 169 for beta-blockers, 170 for ACE inhibitors/ARBs, 469 for P_2_Y12 inhibitors and 206 for attendance to CR

* When adding missing values and reasons of therapy discontinuation, the reported use of P_2_Y12 inhibitors was nearly similar in both groups (85.8% vs. 84.4%). The reported use of P_2_Y12 inhibitors did, however, change over time; clopidogrel from 70.5% to 37.6%, prasugrel from 28.7% to 47.9% and ticagrelor from 0% to 9.2%.

**Fig 2:**
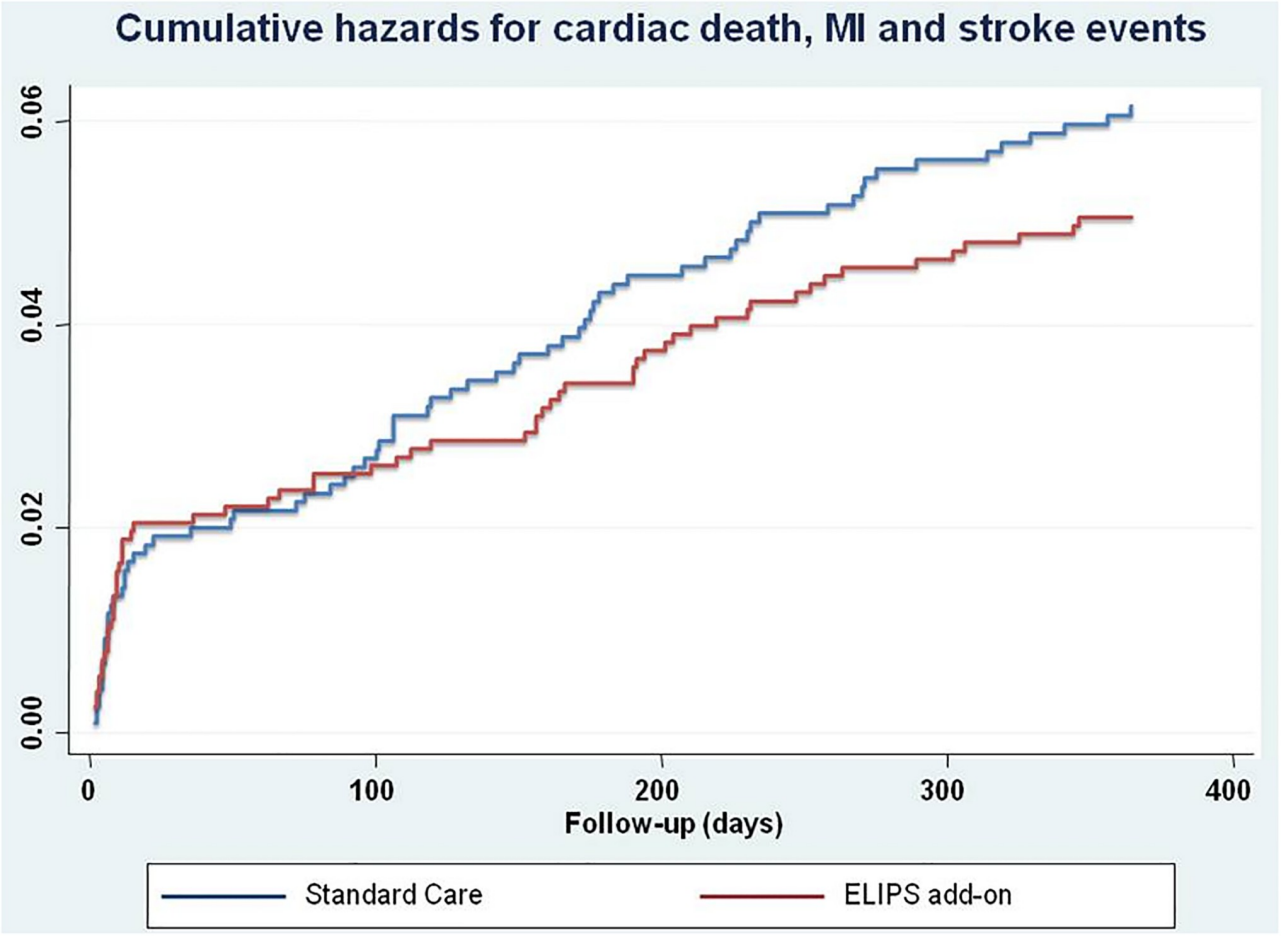
Cumulative hazards for the composite endpoints of cardiac death, myocardial infarction and stroke events over a follow-up period of 365 days in the standard care vs. the ELIPS add-on groups (logrank, P = 0.26). Abbreviations; MI, myocardial infarction.

**Fig 3:**
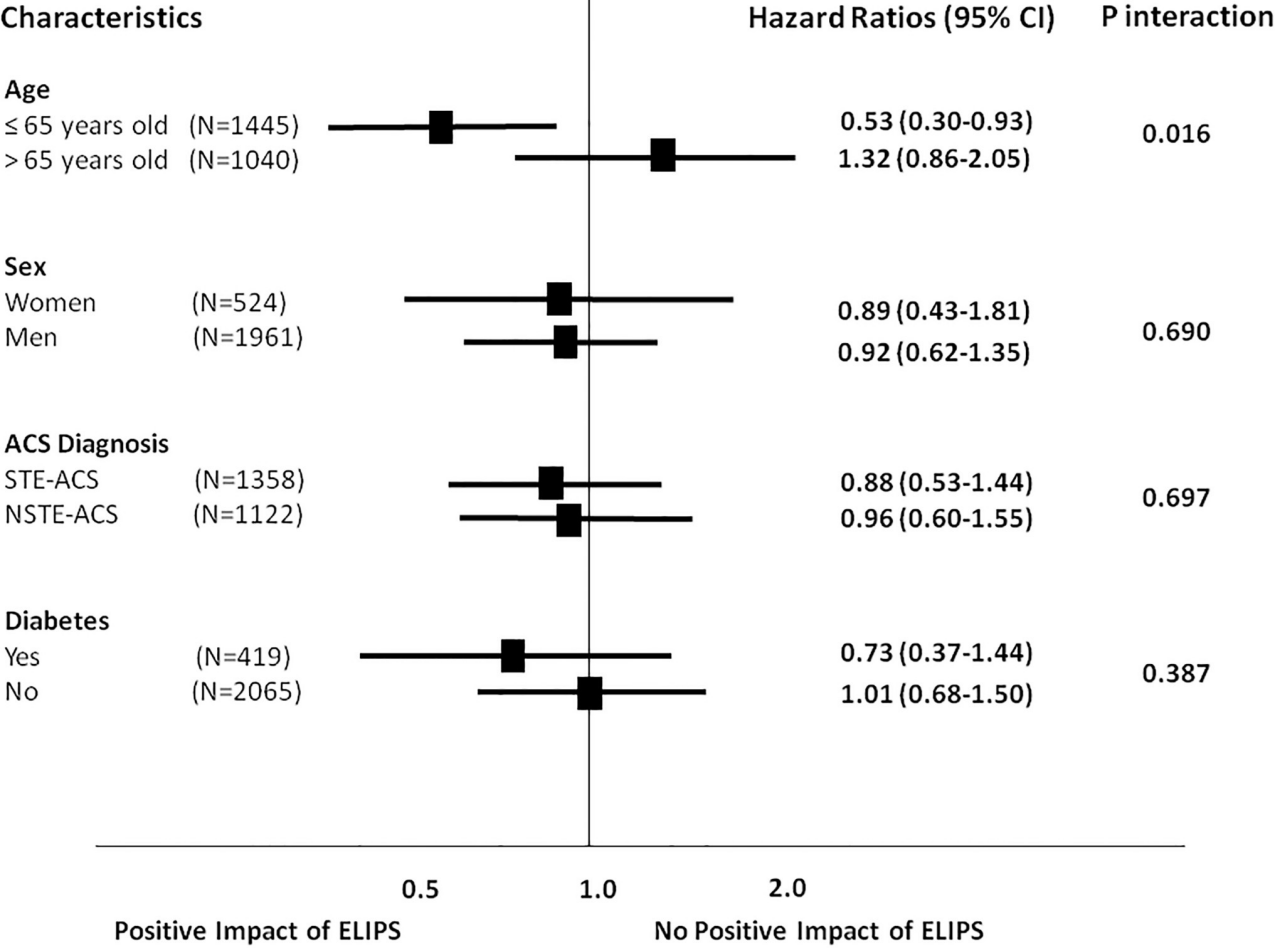
One-year incidence of the composite endpoints of cardiac death, myocardial infarction and stroke events in the standard care vs. the ELIPS add-on groups according to baseline characteristics. Abbreviations: ACS, acute coronary syndromes; CI, confidence intervals; STE-ACS, ST-elevation acute coronary syndromes; NSTE-ACS, non ST-elevation acute coronary syndromes.

The one-year primary endpoint of MACE occurred in 151 patients in the SC group (12.5%) compared to 153 patients (11.9%) in the ELIPS add-on group (age and sex adjusted HR 0.97, 95% CI 0.77–1.22, P = 0.79) (Table 3). No significant differences were found in the incidence of individual outcomes. The cumulative hazards curve for the composite defined by cardiac death, MI and stroke events suggests a trend towards lower events rates in the ELIPS add-on arm vs. SC, especially after hospital discharge (Fig 2). In post-hoc subgroup analyses, significant reductions in cardiac death, MI and stroke events were observed in the ELIPS add-on arm compared to patients ≤ 65 years in the SC group (HR 0.53, 95% CI 0.30–0.93, P for interaction = 0.016, Fig 3); the same observation was made for patients ≤ 55 years (Table 4); there was also a non significant trend towards a higher rate of adverse events in patients above 75 years of age (Table 5). After adjustement for length of stay, the association in patients ≤ 65 year olds was attenuated (HR 0.61 (95% CI 0.35–1.09, P = 0.09), while the interaction was still significant (P = 0.023). After adjustment for cardiac rehabilitation, the effect size of the association for the ELIPS intervention persisted in patients ≤ 65 years, but with a reduction of statistical significance (HR 0.55, 95% CI 0.20–1.49, P = 0.240). Likewise, attendance to cardiac rehabilitation was also nearly associated with significant reductions in cardiac death, MI and stroke events (HR 0.54, 95% CI 0.26–1.14, P = 0.106), but no significant interaction of the ELIPS intervention was found according to clinical rehabilitation attendance (P = 0.897).

**Table 3:** One-year major adverse cardiovascular events and secondary prevention targets in standard care vs. ELIPS add-on groups.

	Standard Care 2009–2010 N = 1210	ELIPS Add-on 2011–2012 N = 1288	Age-sex Adjusted Hazard Ratios (95% CI)	*P*-value
Total MACE*, n (%)	151 (12.5)	153 (11.9)	0.97 (0.77–1.22)	0.79
**Cardiac death, MI and stroke, n (%)**	75 (6.2)	73 (5.7)	0.92 (0.66–1.30)	0.65
**All-cause death, n (%)**	34 (2.8)	29 (2.3)	1.02 (0.62–1.68)	0.95
**Cardiac death, n (%)**	21 (1.7)	17 (1.3)	0.96 (0.50–1.84)	0.91
**MI, n (%)**	39 (3.2)	50 (3.9)	1.14 (0.73–1.78)	0.56
**Stroke, n (%)**	20 (1.7)	13 (1.0)	0.64 (0.30–1.36)	0.25
**Revascularization, n (%)**	82 (6.8)	90 (7.0)	0.99 (0.73–1.35)	0.95
**Hospitalization for unstable angina**	24 (2.0)	29 (2.3)	1.15 (0.67–1.98)	0.61
**Lower limb ischemia, n (%)**	14 (1.2)	12 (0.9)	0.85 (0.39–1.84)	0.68

Abbreviations: CI, confidence intervals; MACE, major adverse cardiovascular events; MI, myocardial infarction

**Table 4:** One-year major adverse cardiovascular events in standard care vs. ELIPS add-on groups in the premature ACS group (N = 779, ≤ 55 years old).

	Standard Care 2009–2010 N = 342	ELIPS Add-on 2011–2012 N = 437	Age-sex Adjusted Hazard Ratios (95% CI)	*P*-value
Total MACE*,** n (%)**	33 (9.7)	39 (8.9)	0.85 (0.53–1.38)	0.97
**Cardiac death, MI and stroke, n (%)**	15 (4.4)	13 (3.0)	0.60 (0.27–1.33)	0.21

Abbreviations: CI, confidence intervals; MACE, major adverse cardiovascular events; MI, myocardial infarction

* Composite endpoints of MACE comprised all-cause death, myocardial infarction, stroke, coronary revascularization, hospitalization for unstable angina, lower limb ischemia.

**Table 5:** One-year major adverse cardiovascular events in standard care vs. ELIPS add-on groups in the elderly group (N = 444, ≥ 75 years old).

	Standard Care 2009–2010 N = 267	ELIPS Add-on 2011–2012 N = 177	Age-sex Adjusted Hazard Ratios (95% CI)	*P*-value
Total MACE*,** n (%)**	51 (19.1)	32 (18.1)	1.00 (0.63–1.55)	0.97
**Cardiac death, MI and stroke, n (%)**	27 (10.1)	23 (13.0)	1.35 (0.76–2.38)	0.30

Abbreviations: CI, confidence intervals; MACE, major adverse cardiovascular events; MI, myocardial infarction

* Composite endpoints of MACE comprised all-cause death, myocardial infarction, stroke, coronary revascularization, hospitalization for unstable angina, lower limb ischemia.

At one year, we noted a significant improvement in the ELIPS group for the achievement of recommended targets, such as LDL cholesterol (74.4% vs 69.8%, RR 1.07, 95% CI 1.02–1.15, P = 0.012), systolic blood pressure (73.4% vs. 66.5%, RR 1.06, 95% CI 1.01–1.13, P = 0.029) and physical activity (54.2% vs. 48.3%, RR 1.10, 95% CI 1.01–1.20, P = 0.024) (Table 6).

**Table 6:** One-year secondary prevention targets in standard care vs. ELIPS add-on groups.

Recommended Targets	Standard Care 2009–2010 N = 1210	ELIPS Add-on 2011–2012 N = 1288	Age-sex Adjusted Rate Ratios (95% CI)	*P*-value
**Biologic parameters**				
LDL cholesterol < 1.8 mmol/l, n (%)	306 (31.9)	361 (35.5)	1.14 (1.01–1.29)	0.037
LDL cholesterol < 2.6 mmol/l, n (%)	670 (69.8)	756 (74.4)	1.07 (1.02–1.13)	0.012
Glucose < 7.0 mmol/l in non-diabetics, n (%)	752 (94.1)	786 (94.4)	1.00 (0.98–1.02)	0.96
Glycated haemoglobin < 7% in diabetics, n (%)	48 (53.3)	73 (56.6)	1.06 (0.82–1.35)	0.67
Systolic blood pressure < 140 mm Hg, n (%)	666 (66.5)	754 (73.4)	1.06 (1.01–1.13)	0.029
Weight reduction≥5% in overweight or obese, n(%)	130 (19.2)	141 (19.4)	1.03 (0.83–1.28)	0.781
**Questionnaire parameters**				
Smoking cessation, n (%)	195 (47.0)	229 (46.8)	0.99 (0.86.1.14)	0.87
Physically active (IPAQ), n (%) *	460 (48.3)	566 (54.2)	1.10 (1.01–1.20)	0.024
Health utility based on EQ-5D, mean (±SD) †	0.81 (0.16)	0.81 (0.17)	NA	0.52
High self-reported adherence (MAS), n (%) ‡	528 (56.3)	582 (54.8)	0.99 (0.92–1.07)	0.80

Abbreviations: EQ-5D, EuroQol-5 dimensions; IPAQ, international physical activity questionnaire; LDL, low-density lipoprotein; MAS, medication adherence scale; NA, not applicable; SD, standard deviation.

* Physically active was defined at least by three days of intense-activity or five days of moderate activity per week.

^†^ Health utility was derived from the EQ-5D questionnaire using European tariffs.

^‡^ High self-reported adherence was defined by a score of 0 on the Morisky Medical Adherence Scale.

Missing values (appropriate or not): 522 for LDL cholesterol, 422 for glucose in non-diabetics, 204 for glycated haemoglobin in diabetics; 469 for systolic blood pressure, 264 for weight reduction, 109 for smoking cessation, 500 for physical activity and 497 for self-reported adherence.

## Discussion

### Main findings

The implementation of the multi-dimensional secondary prevention program after ACS is feasible in a variety of academic centers and is associated with an optimal reported use of educational material during patients’ hospital stay. We did not, however, observe a significant reduction in recurrent cardiovascular events at one year in the overall cohort. In a subgroup analysis, ELIPS (Structured Secondary Prevention Program) patients ≤ 65 years may appear to have gained additional benefit from an educational program, with a reduction in cases of cardiac death, myocardial infarction and stroke events. However, caution is needed in the interpretation of these findings given the *post-hoc* nature of the analysis and the accompanying limitations. The overall neutral result is in part explained by a trend, although not statistically significant, towards a higher MACE rate in patients above 75 years. We also observed an improvement in the control of cardiovascular risk factors, such as the achievement of LDL cholesterol targets, and the level of participation in CR programs was especially high for the ELIPS vs. SC group (72.9% vs. 65.6; p = 0.006), which may be beneficial in the longer term. This finding is especially relevant for the process of care and specifically for the adherence to secondary prevention programs after ACS. Knowing that premature ACS is associated with an important negative impact on quality of life and high opportunity costs, intensive secondary prevention interventions such as ELIPS can be considered of importance in patients with premature ACS, and may be especially interesting for the achievement of favourable long-term outcomes.[19]

### Significance of the ELIPS intervention

Our study is, to our knowledge, the most comprehensive multi-level healthcare quality improvement program performed so far in Europe. When measuring the outcomes of health promotion interventions such as ELIPS, several aspects should be considered: (1) the proportion of patients who actually received the intervention compared to the total pool of eligible patients, (2) the efficacy of the intervention program on clinical and behavioural outcomes, (3) a quantified assessment of adoption uptake of the program by centers, (4) the feasibility of extending the intervention outside the hospital setting, and (5) the sustainability of the program.[20] The ELIPS intervention is characterized by optimal reach, implementation and adoption, but perhaps suboptimal efficacy on clinical outcomes. The long-term integration of educational and quality of care programs into standard care needs to be supported by healthcare decision makers. In Switzerland, the hospital payment system is based on DRGs, which include incentives to shorten the length of hospital stays.[10] Although shortening hospital stays could be considered a barrier for the implementation of hospital-based multidimensional CR programs, standardization of preventive efforts coupled with a well-structured educational program can optimize the process of care for post-ACS patients. The high attendance rate to CR programs observed in our study underlines the importance of implementing secondary prevention early after ACS at the hospital level.[21] In this regard, the ELIPS intervention proved to be effective in encouraging patients to enrol in CR programs. Patients are sensitive to the delivery of preventive messages during their hospital stay and these moments which are focused on education are essential elements of patient-centered care and patient education.[14] Despite these findings, the uptake of CR programs appears to be underused, most likely because of the lack of well-established processes of care.[5] As to the effect that such programs can have on smoking cessation, we observed an approximate 48% cessation rate in the overall cohort, with a remaining 40% of ongoing smokers, which suggests that more specific motivational and medicinal techniques may be required.

### Comparison with previous studies

We have previously shown that the quality of care was especially high in post-ACS patients, with prescription rates reaching up to 100% for aspirin, statins or ACE inhibitors after excluding patients for whom prescription for these medicines was contra-indicated.[17]^,^ [22] The ELIPS add-on intervention was successful in that it left little room for further improvement in the rate of discharge prescriptions and impact on clinical outcomes. For instance, in ACS patients in Brazil, a multifaceted quality improvement intervention including educational materials, reminders, algorithms, and case manager training were associated with a significant increase of adherence to evidence-based therapies.[23] In a meta-analysis that examined a wide variety of secondary programs including education or counselling with an exercise component, a reduction of subsequent MI and mortality was observed,[24] possibly associated with improved clinical outcomes. Another meta-analysis from our group suggested that in-hospital multidimensional secondary prevention interventions after ACS were more effective when including intervention at the provider or system levels, compared with patient-level only interventions.[11] We also previously reported that the most common reason that was provided by patients to justify therapy discontinuation in the out-patient setting was the recommendation received by their own treating physician.[25]. As for the improvement of LDL cholesterol targets observed in the ELIPS add-on phase, this might be explained by the higher rate of participation to CR or the use of high-intensity statin therapy.[26] Regarding the impact of ELIPS on clinical outcomes, the event rate in the observation phase was lower than expected, [18] possibly because of the secular trend towards an overall improvement in the prognosis of post-ACS patients.

### Future outlook

Adherence to national and international guidelines on patient management has been associated with improved post-ACS patient outcomes.[1] Continuous monitoring of performance indicators is strongly recommended by the ESC guidelines in order to minimize the unwarranted variations of quality of care.[4] Several European countries have developed initiatives to improve quality of care according to recommendations and propositions from the ESC (e.g. the Myocardial Infarction National Audit Project [MINAP] registry in the UK). These performance measures were also collected within the EUROASPIRE surveys that reported data on the cardiovascular risk profile in coronary patients.[6] Other initiatives, such as the EUROACTION Study Group, showed that the implementation of local cardiology programs accessible for all hospitals and general practices was feasible.[27] During hospitalization, ACS patients are more likely to be responsive to educational messages and thereby be motivated to follow healthcare recommendations.[16] Therefore, the sustainability of education programs such as the ELIPS add-on intervention is dependent on the support of leaders active in cardiovascular prevention, as well as policy makers responsible for the reimbursement of preventive efforts by hospital-payment systems.

### Limitations

The before-after design is largely used in clinical research aimed at assessing the feasibility/effectiveness of implementing complex interventions into the healthcare system, but it cannot exclude potential confounding factors that might occur during the study.[2, 11] For instance, since 2010 several Cantons in Switzerland have banned smoking in public areas, while at the same time more potent antiplatelet therapies (P_2_Y12 inhibitors) have emerged as treatments of choice. Furthermore, the study sample might not represent all patients hospitalized with ACS (selection bias), as our patients were only included at university hospitals. In fact, not all patients who had cardiogenic shock or undergone resuscitation were included, which perhaps partly explains the low mortality rate in the entire cohort. In addition, study participants were mainly recruited in the catheterization laboratory and might not be representative of those patients not transferred to PCI centers. Although we quantified the utilization of the educational tools used in the ELIPS add-on intervention group, we did not measure the quality of the motivational interviewing, nor did we evaluate whether the educational tools were appropriately used. In fact, we cannot exclude that the two days’ training program on motivational interviewing may not have been sufficient for some care-givers, in particular physicians. Finally, given the non-long term duration of P_2_Y12 inhibitors, the data collection of the variable could be a source of bias according to the date of the follow-up visit, as well as to the cardiologist taking the decision to stop P_2_Y12 treatment.

## Conclusion

The ELIPS program was designed after investigating areas of the healthcare system that offered the greatest potential for improving the quality of care for ACS patients. Although the expected impact on clinical outcomes in the overall cohort was not observed, the implementation of the ELIPS program was associated with a significant improvement in participation in CR programs and control of cardiovascular risk factors. These results should encourage the pursuit of long-term, hospital-based, post-ACS secondary prevention programs.

## Supporting information

S1 Fig Age distribution of the entire study population. (TIF)

S1 Table TREND statement checklist. (PDF)

S1 File ELIPS research protocol. (PDF)

S2 File Dataset. (DTA)
