# Supplementary figures and images for: Clinical impact of a structured secondary cardiovascular prevention program following acute coronary syndromes: A prospective multicenter healthcare intervention
Source: PLoS One. 2019 Feb 21;14(2):e0211464. doi: 10.1371/journal.pone.0211464 (PMC6383891; doi:10.1371/journal.pone.0211464)

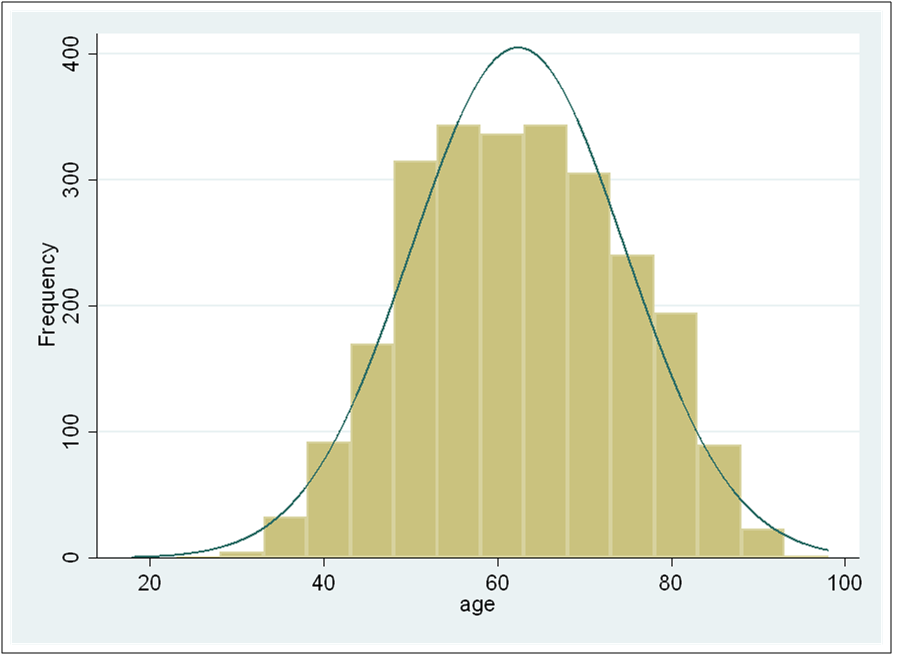

Supplement: S1 Fig — (TIF) [file pone.0211464.s001.tif]
