# Supplementary material for: Clinical impact of a structured secondary cardiovascular prevention program following acute coronary syndromes: A prospective multicenter healthcare intervention
Source: PLoS One. 2019 Feb 21;14(2):e0211464. doi: 10.1371/journal.pone.0211464 (PMC6383891; doi:10.1371/journal.pone.0211464)
Supplement: S1 File — (PDF) [file pone.0211464.s003.pdf]

Protocol 07-131/ February 26 th 2009

MULTI-DIMENSIONAL PREVENTION PROGRAM AFTER ACUTE CORONARY SYNDROME (ELIPS)

Dr Pierre-Frédéric Keller, Dr Nicolas Rodondi, Dr Reto Auer, Prof Thomas Perneger, Prof Alain Golay, M Franck Schneider, Mrs Ariel Richard-Arlaud, Dr Marco Roffi, Dr Sebastian Carballo, Dr David Carballo, Mr Philippe Sigaud, Dr Vincent Barthassat, Dr Christian Matter, Prof Thomas Luscher, Prof Stephan Windecker, Prof Pierre Vogt, Prof Jacques Cornuz, Prof Gérard Waeber, Prof Jean-Claude Chevrolet, Dr Pierre Chopard, Prof François Mach

Table of Content

List of investigators p 3
I) Summary of the research project p 4
II) Summary of the state of research in the field of the research project p 5
The ELIPS programme p 5
III) Research plan of the project p 7
1) Inclusion / Exclusion criteria p 7
a) Inclusion criteria p 7
b) Exclusion criteria p 7
2) Endpoints p 8
3) Data collection and recording on the web-based CRF p 9
4) Obligations for patients in the study p 10
5) Statistical Methods p 10
6) Sample size calculation p 11
7) Project timelines and milestones p 11
8) Novelty and synergies created by this project p 11
IV) Conditions required for hospitals participating in the study p 12
V) References p 14
VI) Annexes p 16
1) Table p 16
2) Web Case Report Form (CRF) p 18
a) CRF at Baseline p 18
b) CRF at 1-year follow-up p 24

Chief Investigator

Dr Pierre-Frédéric Keller
Cardiology Department
University Hospitals of Geneva
Rue Micheli-du-Crest 24
CH 1211 Genève 14
Tel: +4122 3727200
Fax: +4122 3727226
Email: pierre-frederic.keller@hcuge.ch.

List of collaborators

Department of Ambulatory Care and Community Medicine (CHUV)

Dr Nicolas Rodondi
Dr Reto Auer
Prof Jacques Cornuz

Epidemiology Service (HUG)

Prof Thomas Perneger

Service of Therapeutic Education for Chronic Diseases (HUG)

Prof Alain Golay
Dr Vincent Barthassat

Communication / Multimedia Service (HUG)

M Franck Schneider
Mrs Ariel Richard-Arlaud

Cardiology Division (HUG)

Dr David Carballo
Mr Philippe Sigaud
Dr Marco Roffi
Prof François Mach

General Internal Medicine (HUG)

Dr Sebastian Carballo

Cardiovascular Center, Division of Cardiology (USZ)

Dr Christian Matter
Prof Thomas Luscher

Department of Cardiology (Insel Spital)

Prof Stephan Windecker

Cardiology Service (CHUV)

Prof Pierre Vogt

Department of Medicine (CHUV)

Prof Gérard Waeber

Intensive Care Service (HUG)

Prof Jean-Claude Chevrolet

Quality of Care Section, Medical Directorate:

Dr Pierre Chopard

HUG: Hôpitaux Universitaires de Genève. CHUV : Centre Hospitalier Universitaire Vaudois.
USZ : Universität Spital Zürich. Insel Spital : Insel Spital Bern.

MULTI-DIMENSIONAL PREVENTION PROGRAM AFTER ACUTE CORONARY SYNDROME (ELIPS)

I. SUMMARY OF THE RESEARCH PROJECT

Background: Guidelines recommend pharmacologic and lifestyle interventions to reduce recurrence of events in patients with coronary and other atherosclerotic vascular disease. However, audits of practice reveal suboptimal control of cardiovascular risk factors and under use of evidence-based cardiovascular medications. Based on our systematic review of tested interventions, we developed the ELIPS program, a multidimensional secondary prevention program targeting multiple cardiovascular risk factors for patients after an acute coronary syndrome (ACS). This programme targets an increase in prescription rates by physicians and/or long term medication adherence by patients.

OBJECTIVES: To demonstrate the effectiveness of the ELIPS programme (Multi-dimEnsionaL preventIon Program after Acute coronary Syndrome), which aims at improving quality of care of patients admitted to hospital with ACS in the Swiss setting. The program is dedicated to caregivers and to patients to improve their understanding of ACS and atherosclerosis and to increase the motivation for long term treatment.

Methods: A total of 2400 patients will be prospectively included in a multicenter study before and after the implementation of the ELIPS program with a follow-up of 12 months. The primary outcome is a composite of death from any cause, myocardial infarction, documented unstable angina requiring rehospitalization, revascularization (performed at least 30 days after randomization), and stroke. The secondary endpoints are the isolated endpoints of the primary endpoint, and surrogate outcomes such as cardiovascular risk factor control at follow-up. We will also measure process outcome such as improvement in prescription rates of proven-efficacious drug therapies at discharge and at 1 year. A quality monocenter substudy will assess the quality of the information using questionnaires and focus groups techniques.

Expected results: To demonstrate the benefits of the ELIPS program on recurrence rate of cardiovascular events. These results will certainly lead to a generalization of such programs in the field of atherosclerosis in Switzerland and in other countries.

II. Summary of the state of research in the field of the research project

The prognosis of ACS has much improved in recent years as a result of advances in pre-admission and hospital invasive medical management. Nevertheless, the risk of recurrence remains as high as 15% within 12 months[1]. ESC, ACC/AHA Guidelines recommend pharmacologic and lifestyle interventions to reduce recurrent events in patients with coronary and other atherosclerotic vascular disease. In-hospital initiation of evidence-based cardiovascular medications seem to improve long-term patient compliance and clinical outcomes[2, 3]. However, audits of practice reveal suboptimal control of cardiovascular risk factors and under use of evidence-based cardiovascular medication in the US, in Europe as well as in Switzerland[4, 5]. In addition, 30% of patients stop their treatment either partially or totally within 30 days after leaving hospital[6] with a significant increase in 1-year mortality[6]. In the US several projects tested pragmatic interventions targeting an increase in prescription rates by physicians and/or long term medication adherence by patients[7-11]. In Europe, Wood et al recently showed a healthier lifestyle and improvements in cardiovascular risk factors control for patients with coronary heart disease with a nurse coordinated, multidisciplinary, family-based, ambulatory programme[12]. We recently reviewed the evidence of in-hospital multidimensional interventions for patients after an ACS[13]. Secondary prevention programs were categorized as patient-, health care provider-(HCP) and system-level interventions[14]. We found that multidimensional interventions, targeting the patients, the provider and that target an increase in prescription rates of proven efficacious medication seem effective to improve mortality and recurrent ACS[13]. Data from the international GRACE registry show various prescription rates of cardiovascular medication according to regions of the world[15]. Variation across countries might be due to national quality improvement strategies. To date, there is no systematic data collection, national-level quality improvement strategy or incentives to improve care of patients with coronary heart disease in Switzerland. We would like to study the effect of the ELIPS program on clinical and process outcomes in the Swiss setting.

The ELIPS programme:

At the patient-level, ELIPS includes information tools which will be selectively used during well defined steps to respect the appropriateness of the information according to the needs of the patient. Patients will be encouraged to achieve a healthy lifestyle with support from the health professionals who will use motivational interviewing methods throughout the hospital stay[12, 16]. Smokers will have the possibility to enter a smoking cessation program[17], and diabetic patients will be offered a specialized care. The patient will have to self-evaluate their cardiovascular risk factors by the use of an interactive wall chart. We will also gather patients in

interactive round tables in order to improve their understanding and motivation through discussion and interaction. In addition, we will show them a DVD movie as soon as their clinical state will allow it[18, 19]. The film was made after an analysis of the behaviour of ACS patients during filmed interviews, which helped to identify their problems in understanding and their beliefs about ACS and atherosclerosis. At the end of the hospital stay, patients will be discharged with a treatment leaflet which includes the list of the recommended therapies, an explanation of the indications and the targets to achieve in secondary prevention[20]. They will also receive educational brochures. The discharge leaflet will be reviewed by a discharge-planning nurse, who will contact the attending physician if an appropriate medication has not been prescribed or if a contraindication has not been documented[10]. This treatment leaflet will be given to the patient who will transmit it to his family doctor or his cardiologist. A copy will be used as data collection tool that will permit long-term quality measurement for participating hospitals. A dedicated website (visit www.elips.ch) will enable both patients and HCP to further continue their therapeutic education and training respectively. On the health care provider-level, we will identify nurses to receive a two-day educational course on motivational interviewing and cardiovascular health education. All residents and nurses will be offered grand rounds, educational booklets and reminders of the ongoing programme. At a system-level, we will identify a physician champion and a project leader at each hospital[20]. After initial planning, a series of educational sessions will be held to support project implementation. To overcome barriers to implementation, we will include three quality improvement meetings with local project leaders to improve adherence to the ELIPS programme as described in the GAP programme[21, 22]. This will support a collaborative culture of learning and sharing among hospital teams aimed at increasing the use of the AMI standardized tools[22]. The discharge treatment card will permit the development of report cards that will be provided to HCP and hospital administrators.

Follow-up in the outpatient setting will be provided by primary care physicians who will receive a copy of the discharge treatment card, the DVD movie and a presentation of the project. We will also present the project at regional meetings and a national media campaign will inform patients and HCP of the beginning and the aims of the ELIPS programme. All the ELIPS information and teaching tools have been created in several languages and are designed to create a harmonized and professional environment for the education of patients and HCP. (Annexes: Table 1)

III. Research plan of the project

We will study the effectiveness of ELIPS in a multicenter, prospective, controlled before-after study for patients recently hospitalized for ACS. Two thousand four hundred patients will be prospectively included in a multicenter nationwide study before (control group, n=1200) and after the implementation of the ELIPS program (treated group, n=1200) with a follow-up of 12 months. Members of our group have already shown effective recruitment of patients admitted for ACS in the emergency department[23]. We chose a before-after design due to problems of contamination within a hospital (if wards were randomized), difficult randomization of hospitals due to their limited number, and varying functioning and cultures as we will include both French-speaking and German-speaking hospitals. Moreover, many of previous large studies on multidimensional interventions have used such design[13]. The study will proceed in 3 successive phases: In the preparatory phase, we will identify the physician in charge and local program leaders and help them to prepare the outcome measurement tools. In phase 1, we will measure process and clinical outcomes before the implementation of the ELIPS program. We will follow the control patients for 12 months for clinical outcomes. During phase 1, we will help the physicians in charge and local program leaders to prepare the implementation of ELIPS. After this initial phase, we will implement ELIPS during three months. Over a period of 12 months, we will then make the same outcome measurements in intervention patients (phase 2). During phase 2, three quality improvement meetings will help to improve uptake and implementation of ELIPS.

1) Inclusion/Exclusion Criteria

A) Inclusion criteria

All patients >18 years will be prospectively included within 72 hours of presentation in the hospital with the main diagnosis ACS. Any patient admitted for an ACS, that is with symptoms compatible with angina pectoris (chest pain, breathlessness) and at least one of the following 3 features : a) Elevation or depression of the ST segment, inverted T waves or dynamic changes in the repolarization phase*; b) Positive troponin*; c) Known coronary artery disease. *A significant coronary artery disease must be confirmed angiographically.

B) Exclusion criteria

Patients with severe physical disability or dementia will be excluded from study participation. Patients with less than 1 year of life expectancy for non cardiac reason will also be excluded.

ELIPS : Multidimensional interventions and patient education for secondary prevention of ACS

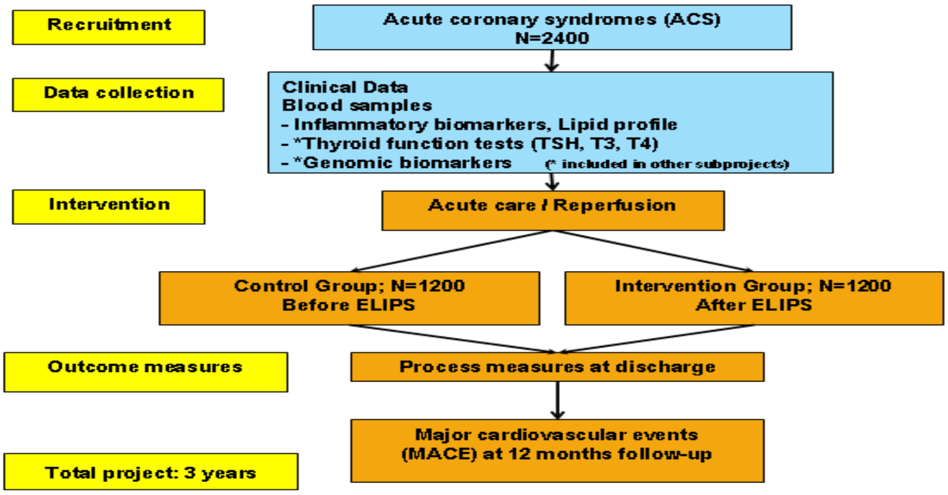

***Biological tests limited to selected centers involved in specifics subprojects.**

2) ENDPOINTS:

All clinical outcomes will be adjudicated by an independent panel of experts with blinding to study groups.

A) PRIMARY (COMPOSITE) ENDPOINT: The primary endpoint will be a composite of death from any cause, myocardial infarction, documented unstable angina requiring rehospitalization, revascularization (performed at least 30 days after randomization), documented new or worsen lower limb ischemia, stroke, and transient cerebral ischemic accident.

B) SECONDARY ENDPOINTS: Secondary outcomes will be individual data on each of these outcomes as well as cardiovascular mortality. We will also measure surrogate outcomes at follow-up such as control of: arterial blood pressure reduction, fasting blood glucose reduction, blood lipids (LDL-Cholesterol reduction, HDL-Cholesterol increase, Triglycerides reduction), smoking cessation, body mass index reduction and abdominal waist reduction.

Definition of the clinical endpoints :

- Sudden death is considered as cardio-vascular mortality as well as fatal myocardial infarction, fatal cerebro-vascular accident of fatal aortic dissection.
- Death as a consequence of vascular or cardiac surgery as well as a lower limb amputation* is considered as cardio-vascular mortality. *Exception: amputation for orthopaedic, cancer, or infection without relationship with a cardiovascular disease or diabetes.
- Death as a consequence of heart failure is considered of cardiovascular origin.

- Unexplained death and / or without apparent cause is considered of cardio-vascular origin (unexplained overnight death) except if a non cardiac cause is proven at autopsy.
- Myocardial infarction is defined as diagnostic criteria of STEMI and NSTEMI defined in the inclusion criteria, taking into account the ESC/ACC/AHA guidelines.
- Myocardial ischemia is defined as the occurrence of angina associated with a proven ischemia at non invasive imaging or a new significant coronary stenosis at angiography.
- Cerebro-vascular attack (or transient ischemic attack) is defined as a diagnostic having required a hospitalization.
- Lower limb ischemia is defined as typical symptoms associated with a diagnostic test (echo-Doppler or angiography).

Methods for measurments of the endpoints :

- Weight (underwear, with an accuracy ≥ 0.1Kg),

- Abdominal waist: horizontal measurement at end-expirium halfway between the iliac peaks and the last coasts with an accuracy of 0.1 cm (2 measurements will be taken and one 3 2nd will be carried out if the difference between 2 measurements is > 0.5 cm and the average of the 2 closest measurements is carried out).

- Blood pressure taken on the left arm : 3 measurements 1 minute from interval with an accuracy of 5 mmHg and the average of 2 last measurements will be used.

c) **PROCESS OUTCOMES:** Prescription rate of evidence based medications will be measured at hospital discharge and at follow-up.

3) DATA COLLECTION AND RECORDING ON THE WEB-BASED CRF

Anonymized demographic and clinical as well as biological data will be collected by local investigators during the index admission and recorded on the baseline case report form (Form 3) available on the following website (http://www.elips.ch) using specific username and password for the local investigators of each hospital. The patient as well as the hospital will be identified by codes. These secure codes allow to the investigators of each hospital to access to their own database. The case report form will be duly completed within 10 days of the patient's discharge. The follow-up medical visit with drawal of a blood sample will have to be done 12 months after the ACS. The clinical and biological data obtained from the clinical visit and from the

blood sample analysis at the follow-up will be recorded on the follow-up case report form (Form 4) on the website (http://www.elips.ch). The blood tests will be analysed in the local laboratory of each hospital site (no laboratory core lab because of the high quality of control of the requested parameters).

The data management Sphinx software is used (Le Sphinx Développement, F-74650 CHAVANOD – France) for the web CRF.

4) Obligations for patients in the study

The conditions and requirements of participation in the present study are as follows:

A medical appointment one year after hospitalization for history, clinical examination and the following measurements: weight, abdominal waist, arterial blood pressure.

A fasting blood sample (15 ml) will be taken at this visit. Measurements will include:

- Fasting glucose
- Total cholesterol
- HDL cholesterol
- Triglycerides
- C-reactive protein

Blood tests will be performed at each site by the certified local laboratory.

5) Statistical Methods

We will first compare the “ELIPS” and control groups in terms of descriptive variables (demographic and clinical baseline variables), using standard tests. If clinically significant differences are detected (whether they are or are not statistically significant), these variables will be used to adjust the comparisons of end-points.

$\chi 2$ tests will be used to compare dichotomous end-points (primary composite event and secondary clinical outcomes). Adjustment for base-line variables will be performed by logistic regression. If the number of adjustable variables is high and the number of events relatively low, we shall employ the technique of “propensity score”: we shall first apply regression analysis to the independent variables to calculate the probability of belonging to the group. We shall then use the predicted probability of this model as an adjuster in the comparison of the groups in terms of primary end-points.

For continuous variables, we shall use linear regression, with the same variable measured in basal conditions as the adjusting variable. Other potentially confounding variables will be added to the model if needed.

6) Sample size calculation

The estimated risk of occurrence of the composite primary clinical outcome in the follow-up period is 15% for the control group[24]. Based on previous studies[13], an acceptable estimate of reduction in risk of occurrence after the intervention is 4% with a reduction in the incidence of the primary outcome to 11% for patients benefiting from the ELIPS programme. Assuming a 2-sided alpha level of 0.05 and a power of 0.80, a sample size of > 1158 patients per group, or about 2300 ACS patients, will be needed to detect this effect size. Based on prior cohorts of elderly patients, such as the Cardiovascular Health Study (CHS), we expect the dropout rate (e.g., from study withdrawal) to be very low (2%) (personal communication from Dr. Alice Arnold from CHS) in these mostly older ACS patients. To account for these potential dropouts, we will increase our sample size by 4% to 2400 ACS patients. To minimize losses to follow-up and maximize retention rate, patients who became housebound or institutionalized and who are unable to go to the hospital for follow-up visits will be visited at home by the study nurse. We will also implement several methods to be able to track all patients (contact information of ≥2 relatives and their primary care physicians, consent to access their hospital records and death certificates, …)[25]. We will monitor patient enrollment and follow-up on an ongoing basis.

7) Project timelines and milestones

- 1st year: preparatory phase and phase 1 will start simultaneously to identify the physician champions and local program leaders and inclusion of patients before the implementation of ELIPS (control group).
- 2nd year: phase 2 for the inclusion of patients after the implementation of ELIPS (treated group).
- 2nd and 3rd years for the medical visit at follow-up, completion of data analysis, manuscript writing and submission.

8) Novelty and synergies created by this project

Having investigated areas in which the greatest impact can still be made to improve the quality of care of ACS patients and based on an in depth analysis of the current literature[13], we have developed the ELIPS programme. This consists of a novel multi-dimensional approach to improve prescription and long term adherence to therapy. The true innovation is the tight collaboration that will be developed and encouraged between all HCP and patients. The full arsenal of multimedia tools and training programme have now been developed and implementation with these novel resources in the context of a nationwide campaign will represent a new federative concept, with the ultimate aim of reducing long term mortality and morbidity associated with ACS.

IV) CONDITIONS REQUIRED FOR HOSPITALS PARTICIPATING IN THE STUDY

1) Approval of the protocol by the local institutional ethic committee.

2) Certification of the accreditation of quality of the chemical laboratory.

3) A training course in therapeutic education will be allocated to 2 members of the medical staff (nurses and physicians) of each participating hospital. Duration of the course: 2 Days with a permanent access to the E-learning on the website www.elips.ch. The course will include include a presentation about:

- The problematic of atherosclerosis: the risk of severe complications of ACS, the risk of relapse of acute cardiovascular events because of the chronic pattern of atherosclerosis,

- The reasons of the use and the process of motivational interviews and brief interventions.

<u>Caregivers training course in motivational interviews:</u>

We plan a two day workshop designed for caregivers coming from collaborating centres. Because caregivers in charge of patients suffering from acute coronary syndrome aren't familiar with chronic disease, this training course aims to make them understand some of the major aspects of chronicity. How can caregivers approach the issue from the patient's perspective? How does this time dimension modify their role? Case-studies will allow the participants to use some theoretical models (Disease acceptance process[26], Transtheoretical model of change[27]) to understand the patient's way of coping with the disease and readiness to change. We will discuss the importance of patient empowerment. We will therefore help caregivers to develop skills in motivational interviewing[28]. The course will include

- The viewing of the film in the conditions described above and instruction as to how it is to be used in therapeutic education.
- The use of the fresco and flyers in the conditions described.
- Instruction as to the inclusion of patients in the Bio-clinical study. The data will be recorded using the Case Report Form questionnaire on the website www.elips.ch.
- Instructions regarding the clinical follow-up of the patient at the end of the 12 months, including the blood test and the data which is to be recorded using the Case Report Form questionnaire on the website www.elips.ch.

4) A training course in the ELIPS program for investigators:

Each principal investigator of hospitals involved in the ELIPS program will be responsible to give regional 60 minutes meetings for outpatient cardiologists and internists in the objectives of the ELIPS program and on the goals of secondary prevention after an ACS.

V) References:

1. Cannon CP, et al. Intensive versus Moderate Lipid Lowering with Statins after Acute Coronary Syndromes. N Engl J Med. April 8, 2004 2004;350(15):1495-1504.
2. Butler J, et al. Outpatient adherence to beta-blocker therapy after acute myocardial infarction. Journal of the American College of Cardiology. 2002;40(9):1589-1595.
3. Smith CS, et al. Early initiation of lipid-lowering therapy for acute coronary syndromes improves compliance with guideline recommendations: Observations from the Orbofiban in Patients with Unstable Coronary Syndromes (OPUS-TIMI 16) trial. American Heart Journal. 2005;149(3):444-450.
4. Fox KAA, et al. Management of acute coronary syndromes. Variations in practice and outcome. Findings from the Global Registry of Acute Coronary Events (GRACE). Eur Heart J. August 1, 2002 2002;23(15):1177-1189.
5. Muntwyler J, et al. National survey on prescription of cardiovascular drugs among outpatients with coronary artery disease in Switzerland. Swiss Med Wkly. Feb 8 2003;133(5-6):88-92.
6. Ho PM, et al. Impact of Medication Therapy Discontinuation on Mortality After Myocardial Infarction. Arch Intern Med. September 25, 2006 2006;166(17):1842-1847.
7. Marciniak TA, et al. Improving the Quality of Care for Medicare Patients With Acute Myocardial Infarction: Results From the Cooperative Cardiovascular Project. JAMA. May 6 1998;279(17):1351-1357.
8. Fonarow GC, et al. Improved treatment of coronary heart disease by implementation of a Cardiac Hospitalization Atherosclerosis Management Program (CHAMP). The American Journal of Cardiology. 2001;87(7):819-822.
9. LaBresh KA, et al. Get With the Guidelines for Cardiovascular Secondary Prevention: Pilot Results. Arch Intern Med. January 26, 2004 2004;164(2):203-209.
10. Lappe JM, et al. Improvements in 1-year cardiovascular clinical outcomes associated with a hospital-based discharge medication program. Annals of Internal Medicine. September 21 2004;141(6):446-453.
11. Eagle KA, et al. Guideline-based standardized care is associated with substantially lower mortality in medicare patients with acute myocardial infarction: The American College of Cardiology's Guidelines Applied in Practice (GAP) projects in Michigan. Journal of the American College of Cardiology. 2005;46(7):1242-1248.
12. Wood DA, et al. Nurse-coordinated multidisciplinary, family-based cardiovascular disease prevention programme (EUROACTION) for patients with coronary heart disease and asymptomatic individuals at high risk of cardiovascular disease: a paired, cluster-randomised controlled trial. Lancet. Jun 14 2008;371(9629):1999-2012.
13. Auer R, et al. Efficacy of in-hospital multidimensional interventions of secondary prevention after acute coronary syndrome: a systematic review and meta-analysis. Circulation. Jun 17 2008;117(24):3109-3117.

14. Ockene IS, et al. Task Force #4--adherence issues and behavior changes: achieving a long-term solution. Journal of the American College of Cardiology. August 21 2002;40(4):630-640.
15. Granger CB, et al. Medication performance measures and mortality following acute coronary syndromes. The American Journal of Medicine. 2005;118(8):858-865.
16. Rubak S, et al. Motivational interviewing: a systematic review and meta-analysis. Br J Gen Pract. Apr 2005;55(513):305-312.
17. Rigotti NA, et al. Interventions for smoking cessation in hospitalised patients. Cochrane Database Syst Rev. 2007(3):CD001837.
18. Krouse HJ. Video modelling to educate patients. J Adv Nurs. Mar 2001;33(6):748-757.
19. Warner L, et al. Effect of a brief video intervention on incident infection among patients attending sexually transmitted disease clinics. PLoS Med. Jun 24 2008;5(6):e135.
20. Mehta RH, et al. Improving quality of care for acute myocardial infarction: The Guidelines Applied in Practice (GAP) initiative. JAMA. March 13 2002;287(10):1269-1276.
21. Montoye CK, et al. A rapid-cycle collaborative model to promote guidelines for acute myocardial infarction. Jt Comm J Qual Saf. Sep 2003;29(9):468-478.
22. Mehta RH, et al. Enhancing quality of care for acute myocardial infarction: shifting the focus of improvement from key indicators to process of care and tool use: The American College of Cardiology Acute Myocardial Infarction Guidelines Applied in Practice Project in Michigan: Flint and Saginaw Expansion. Journal of the American College of Cardiology. 2004;43(12):2166-2173.
23. Wasserfallen J-B, et al. Impact of medical practice guidelines on the assessment of patients with acute coronary syndrome without persistent ST segment elevation. Int J Qual Health Care. October 1, 2004 2004;16(5):383-389.
24. Steg PG, et al. One-year cardiovascular event rates in outpatients with atherothrombosis. Jama. Mar 21 2007;297(11):1197-1206.
25. Hulley SB, et al. Designing Clinical Research: an epidemiologic approach. 2nd ed. Philadelphia: Lippincott Williams & Wilkins; 2001.
26. Thygesen K, et al. Universal definition of myocardial infarction. Journal of the American College of Cardiology. Nov 27 2007;50(22):2173-2195.
27. Prochaska JO, et al. Stages and processes of self-change of smoking: toward an integrative model of change. J Consult Clin Psychol. Jun 1983;51(3):390-395.
28. Miller WR, et al. Motivational interviewing: preparing people for change: Guilford Press; 2002.

VI) ANNEXES:

1) Table: Summary of components of the ELIPS® programme:

A) Training courses and tools which aim at improving the prescription rate of recommended treatments and lifestyle counseling.

	Components	Providers	Time of application
**Educational programme**	Training courses about the treatments of ACS and secondary prevention	- Leader hospitals (CHUV, HUG) of the ELIPS programme for the in-hospital physicians<br><br>- Hospitals involved in the ELIPS programme for the outpatient physicians	Before the beginning of the ELIPS programme
	2-Days Training courses about motivational interviews	- Leader hospital (HUG) of the ELIPS programme	Before the beginning of the ELIPS programme
	Motivational interviews or brief motivational interventions	Medical staff of all hospitals involved in the care of patients with coronary artery disease: ICU, Cathlab, Cardiology division, cardiac rehabilitation	From the admission until the end of the cardiac rehabilitation with encouragement of outpatient consultation in motivational interviews about cardiovascular risk factors
	Swiss media campaign of information	Service of the communication of all university hospitals together involved in the ELIPS programme	Immediately before the beginning of the ELIPS programme
	- Order sets of treatments	Each hospital involved in the ELIPS programme	- During the hospital stay
	- Leaflets of treatment discharge and lifestyle changes for the patient and the outpatient physician	Each hospital involved in the ELIPS programme	- At the discharge of the patient

B) Communication tools which aim at improving the understanding of ACS and atherosclerosis and the therapeutic adhesion of patients.

Tools	Components	Providers	Time of application
**Film (DVD)**	The history of a patient admitted with an ACS: the acute phase and the need of secondary prevention of a chronic disease	- Diffusion: hospital including ICU, Cardiology division and/or cardiac rehabilitation.<br><br>- Distribution: family doctor or cardiologist (campaign of distribution via pharmaceutical delegates)	According to the demand of the patient
**Interactive wall chart**	Information about cardiovascular risk factors, lifestyle counseling and questions, self-assessment of cardiovascular risk factors	Shown in the cardiology division and in the cardiac rehabilitation center	After the discharge of the ICU
**Flyers**	Similar information as on the fresco	ICU, Cardiology division, Cardiac rehabilitation, website	ICU. Cardiology division, cardiac rehabilitation, website
**Website:**<br><br>**www.elips.ch**	- Information about cardiovascular risk factors, lifestyle counseling and questions, self-assessment of cardiovascular risk factors.<br><br>- E-learning in motivational interviews and brief interventions for medical staff.<br><br>- Case Report Form to complete data for the clinical trial using login and password for access of multicentrer and data record	- Hospital medical staff<br><br>- Family doctor<br><br>- Cardiologist	In and out of the hospital

2) Web Case Report Form (CRF):
A) CRF at baseline

ELIPS®
Educational therapeutic program
to fight myocardial infarction
and atherosclerosis

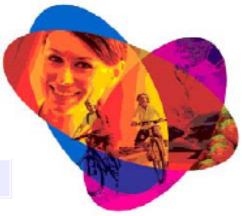

elips-trial@hcuge.ch

**Date of data:**	
**Center ID Number:**	
**Patient ID Number:**	

Baseline Form

Demographic data:

**Subject initial: (first name family name)**		**Date of birth:**		
**Patient treated by ELIPS® program:**	○ Yes	○ No		
**Gender:**	○ Male	○ Female		
**Marital status:**	○ Married	○ Widow(er)	○ divorced	○ Single
**Lives:**	○ Alone	○ With someone		
**Work situation:**	○ Full time	○ Part time	○ No employment	
**Date of hospital admission:**				
**Date of hospital discharge:**				
**Cardiovascular rehabilitation after discharge:**	○ Yes	○ No		
**Which type of rehabilitation:**	○ In hospital	○ Out patient	**Duration of rehabilitation: (weeks)**	
**Specialized out patient consultation about cardiovascular rish factors after the end of cardiac rehabilitation period:**	○ Yes	○ No		

Medical history:

**Family history of coronary artery disease:**	○ Yes	○ No	
**Diabetes:**	○ Yes	○ No	
**Diabetes without complications:**	○ Yes	○ No	
**Diabetes without complications treated**	☐ No treatment	☐ insulin	☐ oral anti-diabetics
**Diabetes with complications:**	○ Yes	○ No	
**Diabetes with complications treated with:**	☐ No treatment	☐ insulin	☐ oral anti-diabetics
**Dyslipidaemia:**	○ Yes	○ No	
**Dyslipidaemia treated:**	○ Yes	○ No	
**Hypertension:**	○ Yes	○ No	
**Hypertension treated:**	○ Yes	○ No	
**Cigarette Smoking:**	○ Current (within past year)<br>○ Never<br>○ Past (stop > 1 year ago)	**Number of packs per day X number of years**<br>*Number of packs per day x number of years*	

History of cardiovascular disease: (before qualifying ACS event)	○ Yes	○ No
Previous myocardial infarction:	○ Yes	○ No
Previous angina without myocardial infarction:	○ Yes	○ No
Prior coronary angioplasty:	○ Yes	○ No
Prior coronary bypass graft:	○ Yes	○ No
Peripheral vascular disease:	○ Yes	○ No
Intermittent claudication, arterial bypass graft, gangrene, acute arterial insufficiency, non treated abdominal or thoracic aneurism (6 cm or more).		
Prior lower limb angioplasty:	○ Yes	○ No
Prior amputation for lower limb arterial insufficiency:	○ Yes	○ No
Prior lower limb arterial graft:	○ Yes	○ No
Prior carotid angioplasty:	○ Yes	○ No
Prior carotid endarterectomy:	○ Yes	○ No
History of cerebro-vascular disease:	○ Yes	○ No
History of stroke with minor or without sequella. If sequella, click hemiplegia under the item: Previous non-cardiac disease.		
History of cerebro-vascular stroke:	○ Yes	○ No
History cerebral transient ischaemic attack:	○ Yes	○ No
Previously treated with aspirin and/or Plavix for cardiovascular disease:	○ Yes	○ No
Previous anti-coagulant treatment for cardiovascular disease:	○ Yes	○ No
Cardiac valvulopathy with cardiological follow-up at intervals < 1 year:	○ Yes	○ No
Symptomatic cardiac valvulopathy:	○ Yes	○ No
Previous heart failure:	○ Yes	○ No
Previous non-cardiac disease:	○ Yes	○ No
Hepatic disease:	○ Yes	○ No
Light hepatic disease:	○ Yes	○ No
Cirrhosis without portal hypertension (ascites) and without bleeding / Chronic hepatitis.		
Moderate or severe hepatic disease:	○ Yes	○ No
Cirrhosis with portal hypertension (ascites) and/or history of gastro-intestinal bleeding from varices.		
Renal disease: (chronic renal insufficiency)	○ Yes	○ No
Moderate or severe renal disease:	○ Yes	○ No
Creatinin > 265 µmol/l or dialysis or renal transplant.		
Pulmonary disease:	○ Yes	○ No
COPD:	○ Yes	○ No
Hemiplegia:	○ Yes	○ No
Resulting from cerebro-vascular disease or other causes (tumour or other). Hemiplegia can be partial or complete.		
Dementia:	○ Yes	○ No
Chronic cognitive deficit.		
Collagenosis	○ Yes	○ No
Systemic lupus erythematosis, polymyositis, Mixed connective tissue disease (MCTD), polmyalgia rheumatica, rheumatoid arthritis, ankylosing spondylarthritis.		

**History of gastric ulcer:**	○ Yes	○ No
Patients having received peptic ulcer treatment, including those who have been cured.		
**Malignant disease:**	○ Yes	○ No
**Leucemia < 10 years:**	○ Yes	○ No
**Neoplasia < 10 years:**	○ Yes	○ No
**Lymphoma < 10 years:**	○ Yes	○ No
**Metastatic neoplasia:**	○ Yes	○ No
**AIDS:**	○ Yes	○ No
All patients with AIDS or ARC.		
**Other(s) disease(s):**	○ Yes	○ No
**If yes, details:**		
**Previous anti-coagulant treatment for non-cardiac disease:**	○ Yes	○ No
**If "yes", treatment > 12 months before present admission:**	○ Yes	○ No

Physical examination:

**Killip class on admission:**	○ Class I	○ Class II	○ Class III	○ Class IV

Class I: No signs of cardiac insufficiency.

Class II: Mild to moderate signs of cardiac insufficiency (S3 gallop, crepitations in lower half of posterior lung fields, or jugular vein distension).

Class III: Significant pulmonary oedema with crepitations above lower half of posterior lung fields.

Class IV: Cardiogenic shock.

**Heart rate on arrival at hospital: (per minute)**			
data from emergency department			
**Arterial systolic pressure on arrival at hospital: (mmHg)**			
data from emergency department			
**Arterial diastolic pressure on arrival at hospital: (mmHg)**			
data from emergency department			
**Height: (cm)**		**le plus proche de l'admission:(kg)**	
Height (cm) nurse data		*en kg selon relevé infirmier*	
**Waist measurement: (cm)**			
Abdominal waist (cm) nurse data			

Timing of angiography and revascularisation :

First cardiac catheterization from admission to hospital to inclusion in the study:	○ None	○ On arrival at the hospital	○ Within 72 hours of admission	○ After 72 hours from admission

Details of revascularization procedure during hospital stay and/or planned:

Angioplasty:	○ Yes	○ No
Date of angioplasty:		
Coronary artery bypass graft surgery:	○ Yes	○ No
Date of coronary artery bypass graft surgery:		
Other cardiac surgery associated with coronary artery bypass graft surgery:	○ Yes	○ No
Aortic valve:	○ Yes	○ No
Mitral valve:	○ Yes	○ No
Tricuspid valve:	○ Yes	○ No
Other cardiac surgery:(VSD...)	○ Yes	○ No
If yes, details:		
Medical treatment only:	○ Yes	○ No

Details of the coronary artery lesions and of the specific intervention:

Significant stenosis of the Left Anterior Descending coronary artery:	○ Yes		○ No		
Treatment of the Left Anterior Descending coronary artery:	☐ Bare Metal Stent	☐ Drug-Eluting Stent	☐ Balloon only	☐ Arterial graft	☐ Vein graft
Significant stenosis of the diagonal branch (one or several branches):	○ Yes		○ No		
Treatment of the main diagonal branch:(if several diagonal arteries are affected)	☐ Bare Metal Stent	☐ Drug-Eluting Stent	☐ Balloon only	☐ Arterial graft	☐ Vein graft
Significant stenosis of the circumflex coronary artery:	○ Yes		○ No		
Treatment of the cirumflex coronary artery:	☐ Bare Metal Stent	☐ Drug-Eluting Stent	☐ Balloon only	☐ Arterial graft	☐ Vein graft
Significant stenosis of the obtuse marginal branch: (one or several branches)	○ Yes		○ No		
Treatment of the main obtuse marginal branch:	☐ Bare Metal Stent	☐ Drug-Eluting Stent	☐ Balloon only	☐ Arterial graft	☐ Vein graft
Significant stenosis of the right coronary artery:	○ Yes		○ No		
Treatment of the right coronary artery:	☐ Bare Metal Stent	☐ Drug-Eluting Stent	☐ Balloon only	☐ Arterial graft	☐ Vein graft
Significant stenosis of the Left Main coronary artery:	○ Yes		○ No		
Treatment of the Left Main coronary artery:	☐ Bare Metal Stent	☐ Drug-Eluting Stent	☐ Balloon only	☐ Arterial graft	☐ Vein graft

Laboratory tests: (normal local values):

Positive troponin before revascularization: ○ Yes ○ No

Maximum CK: (IU/l) (max. level after qualifying event) [ ]

Max. level after qualifying event (IU/l)

Maximum CK-MB: (IU/l) (max. level after qualifying event) [ ]

Max. level after qualifying event (IU/l)

Fasting Blood Glucose: (mmol/l) [ ]

Fasting Total Cholesterol within the first 24h of hospital admission: (mmol/l) [ ]

Fasting tryglicerides: (mmol/l) [ ]

No LDL-Cholesterol calculation if Triglycerides >= 4 mmol/l

Fasting HDL Cholesterol: (mmol/l) [ ]

C-Reactive Protein: (mmol/l) [ ]

First available value

Creatinine: (µmol/l) [ ]

First available value before coronary angiography

Paraclinic tests:

Measurement of left ventricular ejection fraction: (%)

- ○ Preserved:>55
- ○ Slightly reduced:45-55
- ○ Moderatly reduced:30-44
- ○ Severely reduced:<30

(the last measurement carried out during the hospitalization: echocardiography, ventriculography, MRI ou scintigraphy)

ECG persistent ST-elevation at admission > 0.1mV in the standard leads or > 0.2 mV in the precordial leads or new left bundle branch block: ○ Yes ○ No

ST-segment and/or Twave ECG changes other than those mentionned above: ○ Yes ○ No

Normal ECG: ○ Yes ○ No

Complications of the myocardial infarction before initial coronary angiography:

Cardiorespiratory arrest:	○ Yes	○ No
External cardioversion for ventricular fibrillation or haemodynamically unstable ventricular tachycardia:	○ Yes	○ No
Pacing for haemodynamically unstable bradycardia prior to revascularisation:	○ Yes	○ No

Complications of the myocardial infarction after initial coronary angiography:

Bleeding requiring transfusion or emergency surgery:	○ Yes	○ No
Heart failure developing during hospitalization: (Killip III or IV)	○ Yes	○ No
Re-infarction after admission:	○ Yes	○ No
Ventricular arrhythmias > 24 hours after admission:	○ Yes	○ No
Cerebrovascular accident during hospitalization:	○ Yes	○ No
Cardiorespiratory arrest during hospitalization with successful resuscitation:	○ Yes	○ No
Death during hospitalization:	○ Yes	○ No
Acute renal insufficiency requiring dialysis during hospitalization:	○ Yes	○ No

Medication on discharge from hospital:

Total number of different drugs at discharge from hospital (all drugs):	☐	
Aspirin:	○ Yes	○ No
Clopidogrel:	○ Yes	○ No
Other anti-platelet therapy:	○ Yes	○ No
Heparin (unfractionated or low molecular weight):	○ Yes	○ No
Oral anti-coagulant:	○ Yes	○ No
Beta-blocker:	○ Yes	○ No
ACE inhibitor:	○ Yes	○ No
Angiotensin II receptor antagonist:	○ Yes	○ No
Spironolactone / Epleronone:	○ Yes	○ No
Calcium antagonist:	○ Yes	○ No
Nicorandil, Molsidomine or long-acting nitrates:	○ Yes	○ No
Digoxin:	○ Yes	○ No
Diuretics:	○ Yes	○ No
Statins:	○ Yes	○ No
Ezetimibe:	○ Yes	○ No
Other hypolipidaemic:	○ Yes	○ No
Amiodarone:	○ Yes	○ No
Antidepressants:	○ Yes	○ No
Sedatives or tranquilisers:	○ Yes	○ No

Results of calculations:

Duration of hospitalization:	☐
Body Mass Index:	☐
Charlson's score	☐
LDL Cholesterol: (mmol/l)	☐

B) CRF at 1-year follow-up

ELIPS®
Educational therapeutic program to fight myocardial infarction and atherosclerosis

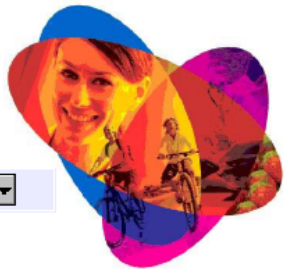

elips-trial@hcuge.ch

Date of data:

Center ID Number:

Follow up Form

Patient ID Number:

Demographic data:

**Subject initial:(first name family name)**	**Date of birth:**	**Gender:** ○ Male ○ Female

**Death between discharge from hospital and 12 month follow-up:**	○ Yes	○ No

**Ethiology of death:**	○ Cardiovascular	○ Other	○ Unknown

Sudden death is considered to be cardiovascular as are fatal myocardial infarct, fatal cerebrovascular accident or fatal aortic dissection. Death following vascular or cardiac surgery or an amputation which is not due to an orthopaedic or tumoral problem or to an infectious cause which itself is not related to vascular disease or diabetes.Death consequent on heart failure
Death which is unexplained or has no apparent cause is considered to be cardiovascular (nocturnal unexplained death) unless a non-cardiac cause is revealed at autopsy.

History obtained from the patient at 1 year :

**Diabetes:**	○ Yes	○ No
**Treated by insulin:**	○ Yes	○ No
**Treated by oral anti-diabetics :**	○ Yes	○ No
**Dyslipidaemia:**	○ Yes	○ No
**Dyslipidaemia treated :**	○ Yes	○ No
**Hypertension :**	○ Yes	○ No
**Hypertension treated:**	○ Yes	○ No

**Cigarette smoking:**	○ Current smoker	○ Ex smoker	**Number of packs per day X Number of years:**

**Smoking stopped since initial hospital admission:**	○ Yes	○ No
**Smoking stopped before initial hospital admission:**	○ Yes	○ No

Recurrence of cardiovascular event since initial hospital admission:

○ Yes ○ No

Myocardial infarction:

○ Yes ○ No

Myocardial infarct is defined according to diagnostic criteria bringing together STEMI and NSTEMI when a hospital admission has resulted according to international ESC/ACC/AHA recommendations.

Recurrent coronary ischaemia excluding myocardial infarction:

○ Yes ○ No

Myocardial ischaemia is defined by the appearance of recurrent angina with a record of ischaemia on imaging or by the appearance of a significant new coronary artery stenosis with or without revascularisation.

Occurrence of cerebrovascular accident:

○ Yes ○ No

A cerebrovascular accident or transient cerebral ischaemia are defined by diagnoses which have resulted in hospital admission.

Occurrence of transient cerebral ischaemia:

○ Yes ○ No

A cerebrovascular accident or transient cerebral ischaemia are defined by diagnoses which have resulted in hospital admission.

Development of lower limb ischaemia:

○ Yes ○ No

Lower limb ischaemia is defined by typical symptoms in association with a diagnostic test (ultrasound or angiography).

Development of heart failure:

○ Yes ○ No

Revascularisation treatment since discharge from hospital:

Revascularisation treatment since discharge from hospital:

○ Yes ○ No

Treatment:

☐ Angioplasty ☐ Coronary Artery Bypass Graft surgery associated with non-coronary cardiac surgery

☐ Coronary Artery Bypass Graft surgery ☐ Medical treatment alone

If angioplasty:

○ Restenosis only ○ Worsening of atherosclerosis with new lesions only ○ Restenosis and worsening of atherosclerosis with new lesions

If Coronary Artery Bypass Graft surgery :

○ Restenosis only ○ Worsening of atherosclerosis with new lesions only ○ Restenosis and worsening of atherosclerosis with new lesions

Physical examination:

Heart rate at 12 months (per minute):

Arterial systolic blood pressure at 12 months:

Arterial diastolic blood pressure at 12 months:

Blood pressure in the left arm: 3 measurements at 1 minute intervals with accuracy of 5 mm Hg. The mean of the 2 later measurements will be used.

Poids en kg à 1 an:

Weight (in underclothes, accurate to 0.1 kg).

Waist measurement (cm):

Waist: measured horizontally in end expiration, mid way between the iliac crests and the lowest ribs with an accuracy of 0.1 cm (2 measurements are to be made and a 3rd if the difference between the first 2 is > 0.5 cm. Then the mean of the 2 closest values will be taken).

Present medication: (all drugs)

Total Number of different drugs at 1 year (all drugs):		
Cardiovascular medication at 1 year:	○ Yes	○ No
Aspirin	○ Yes	○ No
Clopidogrel	○ Yes	○ No
Other antiplatelet therapy:	○ Yes	○ No
Heparin (unfractionated or low molecular weight):	○ Yes	○ No
Oral anti-coagulant:	○ Yes	○ No
Beta-blocker:	○ Yes	○ No
ACE inhibitor:	○ Yes	○ No
Angiotensin II receptor antagonist:	○ Yes	○ No
Spironolactone / Epleronone:	○ Yes	○ No
Calcium antagonist:	○ Yes	○ No
Nicorandil, Molsidomine or long-acting nitrates:	○ Yes	○ No
Digoxin:	○ Yes	○ No
Diuretics:	○ Yes	○ No

Statins:	○ Yes	○ No

Statins dosage:	○ No change	○ Increase	○ Decrease

Change of statins:	○ Yes	○ No
Ezetimibe:	○ Yes	○ No
Other hypolipidaemic agent:	○ Yes	○ No
Amiodarone:	○ Yes	○ No
Antidepressants:	○ Yes	○ No
Sedatives or tranquillisers:	○ Yes	○ No

Laboratory tests (normal local values):

Fasting Blood glucose (mmol/l):

Fasting Total Cholesterol (mmol/l):

Fasting Triglycerides (mmol/):

Fasting Cholesterol HDL (mmol/l):

Fasting Cholesterol LDL (mmol/l):

No LDL Cholesterol calculation if Triglycerides >= 4 mmol/l

C-Reactive Proteine (mmol/l):
